# Views of Pharmacists and Government Representatives Toward the Pilot Chief Pharmacist System in Chinese Hospitals: A Multicenter Exploratory Qualitative Study

**DOI:** 10.3389/fpubh.2022.895649

**Published:** 2022-06-15

**Authors:** Ruomeng Yang, Qian Li, Khezar Hayat, Panpan Zhai, Wenchen Liu, Chen Chen, Amna Saeed, Jie Chang, Pengchao Li, Qianqian Du, Sen Xu, Jun Wen, Yu Fang

**Affiliations:** ^1^Department of Industrial Economics and Trade, School of Economics and Finance, Xi'an Jiaotong University, Xi'an, China; ^2^Department of Pharmacy Administration and Clinical Pharmacy, School of Pharmacy, Xi'an Jiaotong University, Xi'an, China; ^3^Center for Drug Safety and Policy Research, Xi'an Jiaotong University, Xi'an, China; ^4^Shaanxi Centre for Health Reform and Development Research, Xi'an, China; ^5^Institute of Pharmaceutical Sciences, University of Veterinary and Animal Sciences, Lahore, Pakistan

**Keywords:** chief pharmacist system, qualitative study, China, pharmaceutical service, health system

## Abstract

**Background:**

In China, the pharmacy departments of most hospitals have changed their main focus from drug procurement and distribution to providing pharmaceutical care services. Various regions of China have successively implemented the pilot Chief Pharmacist System (CPS) to help improve pharmaceutical care services and rational drug use in hospitals. This study was designed to explore the perspectives of pharmacists and government officials on CPS, including the advantages and barriers to the successful implementation of CPS.

**Methods:**

A qualitative study, based on semi-structured interviews, was conducted from October 1, 2018 to March 1, 2019. The interview data were gathered from 18 pharmacy staff and government representatives working in five distinct regions of China using purposive sampling. A thematic analysis approach and NVivo version 12 Plus was utilized to code and analysis of all interviews.

**Results:**

Five broad themes were identified: the role of the chief pharmacist; their attitudes toward the CPS; the advantages and results of the CPS; the barriers toward CPS; and their suggestions toward CPS. Most of the participants believed that the chief pharmacist played a vital role in a hospital. Under CPS, the hospital pharmacy department pays more attention to prescription review, medication monitoring, and pharmaceutical consultation. However, an insufficient number of pharmacy personnel, unclear authority, and inadequate salaries were the main barriers to the implementation of the CPS.

**Conclusion:**

The attitudes of most of the participants were found to be positive toward CPS in China. The CPS can enhance the prestige of the hospital pharmacy department, improve the quality of hospital pharmaceutical care services, and promote rational drug use. Nevertheless, certain barriers highlighted in this study should be addressed promptly.

## Background

According to the European Association of Hospital Pharmacists (EAHP), the fundamental role of the hospital pharmacy service is to “optimize patient outcomes, by collaborative working within multidisciplinary teams to achieve judicial use of medicines” (1–4). The hospital pharmacy department is an important pillar, and the hospital pharmacist plays a crucial role in the rational use of drugs (5–7).

In China, the pharmacy department of most hospitals has not yet got true recognition in terms of pharmaceutical care services as seen in developed countries like the US, United Kingdom and Australia. Most of China's hospital pharmacies are still primarily focused on providing traditional drug-based services, such as drug supply and inventory management. However, their patient-centered roles are still in the evolving phase (8–11). Under these circumstances, the roles, responsibilities, and status of hospital pharmacists in rational drug management have been neglected for a long time. Therefore, the true value of pharmacists cannot be exerted (12–16). In 2009, the public hospitals in China implemented the Zero-markup drug policy, in which essential drugs are sold to patients for the procurement price plus a predetermined distribution cost, with no profit to the health institution. This policy highlighted the significance of the pharmaceutical department in improving patient affordability and stimulated further reforms in this department (17–22). In 2018, Chinese hospital pharmacy departments have successively implemented the pilot Chief Pharmacist System (CPS) in numerous regions of China under the vigorous promotion of the National Health Commission with the aim to enhance rational drug use and offer pharmaceutical care services to the patients (23, 24). With the implementation of CPS, Chinese hospital pharmacists were empowered to play their vital role in providing pharmaceutical care-related services to patients as a leader. There were three key strategies of CPS, including (1) the establishment of performance indicator system for rational drug use coupled with rewards in terms of financial incentives for those who meet the standards, (2) shifting the focus of pharmacists from drug product to patient in order to enhance their engagement in offering pharmaceutical care services, (3) monitoring and review of rational drug use by establishing an interdisciplinary team involving key stakeholders of healthcare system.

Traditionally, the head of a pharmacy department was not given the power and responsibility to manage all aspects of hospital pharmacy, leading to chaotic management of the hospital pharmacy department. So, according to CPS, an experienced pharmacist with both professional and managerial skills was elected chief pharmacist who became in charge of the development of hospital pharmacy. Chief pharmacists needed to perform their management roles including drug supply management, rational drug use guidance in clinical departments, prescription review, and provision of pharmaceutical care. The objective of CPS was to bring reforms to the current health system. This would allow hospital management to improve the level of pharmaceutical services and provide safe, effective and economic treatment to people (25–30).

By the end of December 2018, the first batch of hospitals in China that had carried out pilot CPS included 64 hospitals, among which 24 hospitals were managed by the Beijing Municipal Hospital Authority, 10 hospitals were in Baoji city, 23 hospitals were in Ankang city, (Shaanxi Province), 3 public hospitals were in Qingdao, Shandong Province, and 4 tertiary public hospitals were in Xinjiang Uygur Autonomous Region and the medical group in Shenzhen Luohu District (30–35). The pilot areas adopted the CPS differently, according to their circumstances. Baoji city, Ankang city of Shaanxi Province, Qingdao city of Shandong Province, and the Urumqi city of Xinjiang all set up chief pharmacists at the hospital level who was responsible for the management of the whole hospital pharmacy department. Whereas, the Beijing municipal authority had set up a team of chief pharmacists that focused on seven different working directions including drug supply and quality management, rational drug use and drug safety management, pharmaceutical care service, training of healthcare professionals, scientific research, and quality management of Chinese medicine. The chief pharmacist team managed the development of pharmaceutical care services in 22 hospitals operated by the authority. The Luohu Medical Group was selected to implement the CPS in Shenzhen and Guangdong provinces. The chief pharmacists were selected at the medical group level to manage the development of pharmaceutical care services (27–33, 35, 36).

As CPS is a new model, which has been implemented in a few selected hospitals, there are only a few studies in this field at present. *Expert Consensus on Chief-Pharmacist System in China* published in October 2018 described the CPS comprehensively for the first time (37). Another study evaluated drug expenditures and medical service quality during the pre- and post-implementation periods and found that CPS could significantly reduce drug expenditures and promote rational drug use (38). However, there is no research literature based on qualitative study design, involving semi structured interviews to inquire deeply into the perspective of pharmacists and government personnel on the CPS. Therefore, our objective was to determine the views of the chief pharmacists and government representatives on the implementation of the CPS in Chinese hospitals, including its advantages and barriers.

## Methods

### Study Design

A qualitative study design based on in-depth interviews was used to investigate the perspective of pharmacists and government officials on CPS. This study design is of great importance when a study is exploratory or aimed to collect attitudinal information. Moreover, this type of study design is flexible and provides useful insight into the experiences, intentions, and attitudes of the participants (39). Meanwhile, the interviewer can use probing questions to get a complete picture of a particular scenario according to the study questions (40).

### Study Settings

This study was carried out in five distinct regions of China, including Shaanxi (western region), Xinjiang (western region), Shandong (eastern region), Guangdong (southern region), and Beijing (northern region) (41, 42). These regions were selected because they were the only areas where the CPS was implemented before December 2018.

A random sample of hospital was drawn proportionally to the percentage of hospitals implementing CPS at each region. Both tertiary and secondary hospitals were randomly selected from the Shaanxi Province (three tertiary and one secondary hospitals), only tertiary hospitals were selected from Xinjiang (three), Shandong (three) and Beijing (four), and only secondary hospitals were selected from Guangdong (two). In total, 16 hospitals were selected to recruit pharmacists (chief pharmacists or heads of pharmacy departments). Moreover, the representatives of the provincial health committee of each province were also invited to this study.

### Development of the Interview Guide

A thorough literature review was conducted to develop a reliable interview guide (5, 27, 43–46). Two qualitative study design experts reviewed the interview guide to determine its validity, and face-to-face interviews assured its reliability. The study was piloted on two participants who were not included in the final data analysis. Only minor changes were made to the interview guide as the questions were according to the experience of the participants, and they were able to understand them easily. The interview guide consisted primarily of four parts, including the responsibilities of the chief pharmacist, rational drug use, pharmaceutical care services and experience in the implementation of the CPS (5, 44, 47, 48).

### Enrollment of Participants

The purposive sampling method with the snowball technique was used to enroll the participants in this study (49). A formal invitation was sent telephonically to all participants and once they agreed, they were interviewed by a trained researcher (QL). All interviews were conducted in Chinese language and then translated into the English language for analysis. An expert committee performed forward and backward translation to ensure the accuracy of the translation. Only the heads of the pharmacy department, the chief pharmacist, and government representatives were included in this study. All interviewees were experts in the field of drug policy and have extensive work experience. Most of them were directly involved in the implementation of CPS and were familiar with CPS.

### Ethics Statement

The study was approved by the Ethics Committee of Xi'an Jiaotong University (number: 2019-1243). Written and verbal consent was obtained from all the participants. They were informed of their right to withdraw at any stage from the interview as their participation was voluntary.

### Data Analysis

All interviews were deidentified, recorded audio and transcribed verbatim. A thematic analysis was performed by a research team to identify themes and sub-themes by following the steps described elsewhere (50–52). A qualitative data management software (NVivo version 12 Plus, QSR International, Melbourne, Australia) was utilized to code all the interviews by two researchers (KH and QL). Once the coding process was completed, a comparison was made to ensure agreement. After repeated discussions, the coding was finalized. At the 16th interview, the saturation point (a point after which no new theme emerges) was achieved (53, 54).

## Results

Out of the total (18), 11 participants were chief pharmacists (61.1%), five were the director of the pharmacy department (27.8%) and two participants were government representatives (11.1%). Most of the participants were between 40 and 50 years of age (61.1%) and most of the participants had a post-graduate degree (88.89%). Most of the participants had more than 20 years of working experience (77.8%) (Table 1).

**Table 1:** Demographic information of participants (*n* = 18) and hospitals characteristics (*n* = 16).

**Characteristics**	**Total *n* (%)**
**Age (years)**	
30–40	1 (5.6)
40–50	11 (61.1)
>50	6 (33.3)
**Gender**	
Male	8 (44.4)
Female	10 (55.6)
**Experiences (years)**	
10–20	4 (22.2)
20–30	12 (66.7)
>30	2 (11.1)
**Education**	
Bachelor	2 (11.1)
Master	9 (50.0)
PhD	7 (38.9)
**Position**	
Chief Pharmacists	11 (61.1)
Director of the pharmacy department	5 (27.8)
Government staff	2 (11.1)
**Bed capacity of hospitals (*****n*** **=** **16)**	
<1,000	6 (33.3)
1,000–2,000	5 (27.8)
>2,000	7 (38.9)
**Hospital level (*****n*** **=** **16)**	
Tertiary hospital	13 (81.2)
Secondary hospital	3 (18.8)

**Figure 1:**
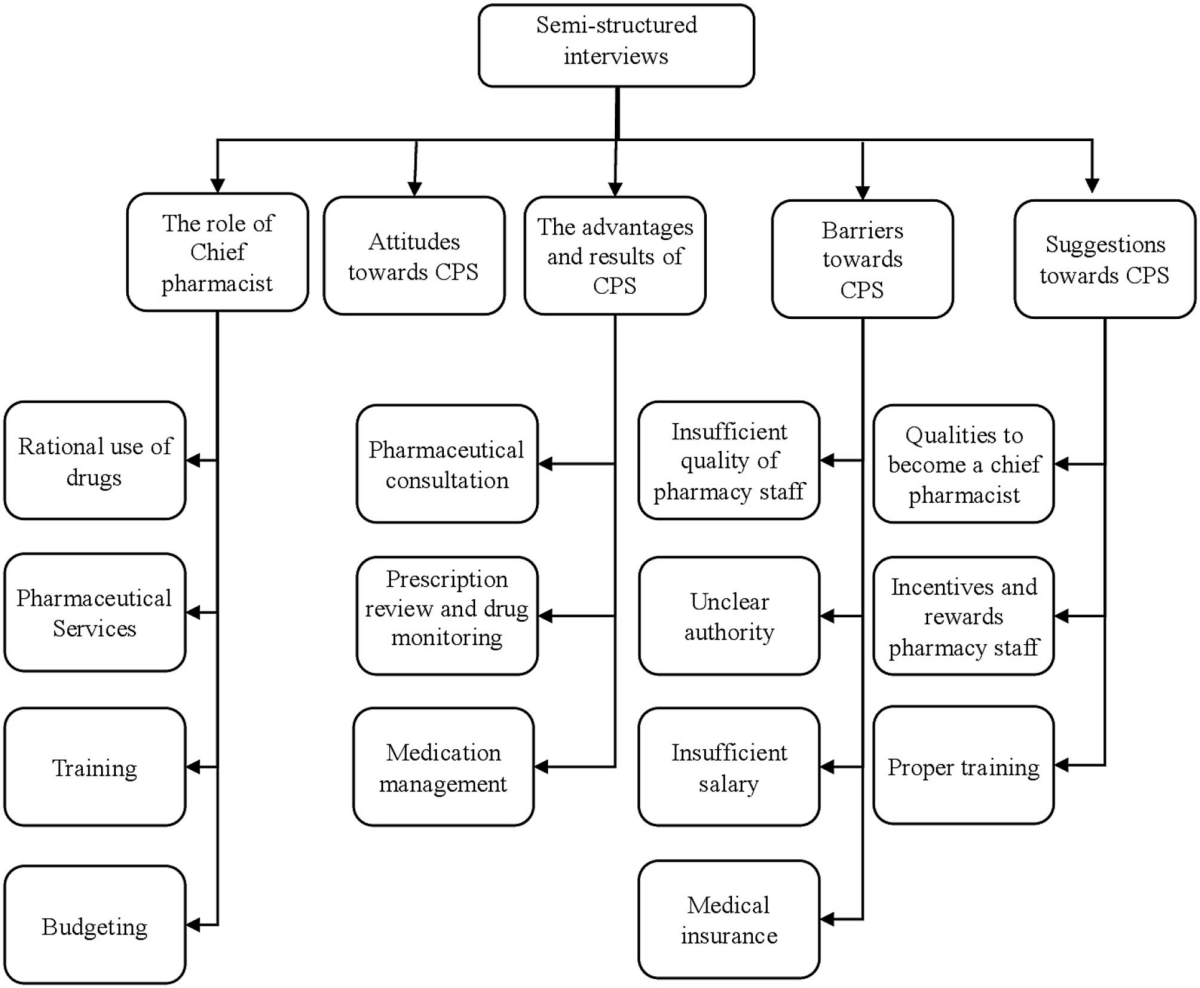
Summary of emerged themes and subthemes.

The themes that emerged from the semi-structured interviews were broadly classified into five major categories: (1) The role of chief pharmacist; (2) Their attitudes toward the CPS; (3) The advantages and results of the CPS; (4) The barriers toward CPS; (5) Their suggestions toward PS (see Figure 1).

### Role of Chief Pharmacist

#### Rational Use of Drugs

Almost all participants reported that the chief pharmacist can play an important role in the rational use of the medication.

“*What our chief pharmacists have to do with the help of clinical pharmacists is to review prescriptions to determine the rationality of the prescribed drugs. We have now increased the intervention rate, which requires the pharmacist to meet with the doctor every month to tell them the prescription problems directly; the purpose is to improve the prescribing pattern of physicians.” (P4)*

“*We hold a special meeting on rational drug use in every quarter to half a year.” (P5)*

The participants also indicated that some penalties that need to be imposed on the pharmacists if they are unable to meet the standard guidelines for rational drug use.

“*If the usage and dosage of the medicines are unreasonable, they would be fined two thousand yuan.” (P1)*

#### Pharmaceutical Care Services

Some participants believed that the chief pharmacist played an important role in improving the overall hospital pharmacy services. Hospitals that have implemented CPS had taken relevant measures to improve pharmaceutical care services.

'*We have created a specialist team, consisting of pharmacists who can work in pharmaceutical clinics to provide services related to pharmaceutical care such as counseling.' (P7)*

“*……., we need to upgrade the patient counseling center for better pharmacy service, and we have established uniform standards for the drug consultation. This center has adequate space and relevant staff. Only a qualified person can work in these centers.” (P9)*

“*We have a medication education service such as on-site counseling for in-patients at the pharmacist's clinic to provide complete drug and disease-related information to patients.” (P12)*

#### Training

The chief pharmacist has also emphasized training hospital pharmacists to equip them with the latest medical information. Under the leadership of the chief pharmacist, various pilot hospitals have launched a series of training activities for pharmacists.

“*We use an hour to train pharmacists every Tuesday. Through a study of several months, everyone feels that this professional training is better combined with clinical cases. In addition, there are more opportunities for out-of-office training. This year, the training program was held more than 40 times, and numerous pharmacists working in different departments of the hospital participated.” (P4)*

'*We have also paid attention to scientific research, as well as to pharmacist training.' (P6)*

'*The chief pharmacists have played a key role in designing and conducting numerous professional training programs for pharmacists to optimize the provision of pharmaceutical care services.' (P9)*

#### Budgeting

Some participants indicated that the chief pharmacist was also responsible for the procurement of drugs and budget management.

“*To verify the drug procurement plan, our hospital selects drugs according to the principle of well-known manufacturers that offer pharmaceutical products at a reasonable price. To reduce the economic burden of patients, the use of imported drugs is discouraged as they are expensive however their availability in the hospital is ensured.” (P5)*

“*The chief accountant is responsible for the financial management of the entire hospital. We give a budget at the end of each year. Then, a summary analysis of the procurement is carried out regularly.” (P4)*

#### Attitudes Toward the Chief Pharmacist Policy

All participants accepted that the implementation of the CPS in different hospital settings in China could improve drug utilization.

“*We are in favor of this (CPS) system. It is like the new medical reform; everyone is talking about it now. So, at this time, I think it is very timely and necessary to emphasize this pilot work. I feel blessed to be a part of this pilot project, and a lot of pharmacists in my hospital are appreciating this effort.” (P14)*

“*Establishing the Chief Pharmacist System is good. I think it is helpful to use this (CPS) to improve the value and prestige of pharmacists so that they can do more, and it must be helpful to let pharmacists play a role that society continues to recognize. This is indeed very worthwhile.” (P15)*

“*I think the Chief Pharmacist System is developed to improve the level of drug utilization. If the Chief Pharmacist System is not implemented, it is challenging for pharmacists to do it on an individual basis. Now, due to the implementation of the Zero Markup Policy, the salary of pharmacists has been reduced. Additionally, there is a shortage in medical insurance funds, coupled with inadequate pharmaceutical care services. It is the right time to implement the Chief Pharmacist System in China. This will not only provide benefits to the pharmacy personnel in terms of their salary and authority but also improve the level of drug treatment.” (P16)*

#### Advantages of the CPS

A large number of participants reported that CPS has many advantages, and positive results have appeared in many aspects within the hospital.

#### Pharmaceutical Consultation

The implementation of the CPS has made improvements in drug consultations in hospitals. Some hospitals have solved the problem of pharmacist consultation fees and some hospitals have added judicious drug consultation services.

“*We solved the problem with the pharmacist consultation fees. From the beginning of last year, I met clinical pharmacists and solved the problem of the consultation fee. Now, like doctors, the consultation fee for the pharmacist is 26 RMB in our hospital, and for the deputy pharmacist, the consultation fee is 13RMB, which is the same as the clinical consultation fee. The maximum number of consultations that could be provided is almost 40 per month. The main content is to discuss the rational use of antibacterial drugs. Additionally, the clinical pharmacists have also participated in the discussion of difficult and critical cases in the hospital.” (P4)*

“*Establish a rational drug consultation office. Consultation is conducted in the outpatient clinic every week, more than 3 times a week, and now it is carried out almost every day. They (pharmacists) mainly answer the problem of medication to patients with chronic diseases and specific diseases and counsel them regularly. The cases of the patient took the initiative to consult the pharmacists less than 10 cases per day, indicating that the patient's habit of consulting the pharmacist has not yet been developed.” (P5)*

#### Prescription Review and Drug Monitoring

The participants believed that after implementing the CPS, hospitals would help pharmacists participate in the prescription review process, along with feedback to help improve physician prescribing habits.

'*Communication with clinical practitioners has improved further after the implementation of CPS. Now, with the introduction of the prescription review management method, the prescription review function of our dispensing pharmacists has also been strengthened. The prescriptions of seven to ten days per month are used for this review process, and the results of the review are reported on the official WeChat group as feedback to improve the rational drug use.” (P4)*

'*We conduct prescription reviews every month, comment on the phenomenon of irrational drug use, and propose corrective measures. The response is outstanding. Focus on monitoring the top 20 drugs and conduct statistical analysis every quarter. It is safe, effective, economical, and adaptable to the choice of drug quality and drug treatment.' (P5)*

'*After the prescription evaluation, we hold a meeting to train the director of the pharmacy department and the pharmacists responsible for the prescription review. The content includes how to improve the prescription pass rate and what you want everyone to do. Every time I analyze the results of these prescription reviews, I inform hospitals with the most prescription-related problems to improve their prescriptions, so next time they can improve step by step.' (P9)*

#### Medication Management

Some participants indicated that hospitals put more emphasis on the management of chronic diseases by pharmacists and provided multiple pharmacy services to promote the management of medications in such cases.

“*……., we have launched a special project for chronic disease management. For example, in diabetes management, medication guidance is offered to patients that facilitate their self-management, which could help improve the general management of chronic diseases. We have also established a national clinical pharmacist training program this year' (P12)'*

“*……….. our pharmacist's service was mainly in the pharmacy. However, with the reforms of medical institutions, now our pharmacist's service has gone out of the pharmacy and reached into the patient wards, by providing medication consultations and formation of pharmacy clinics. Pharmacists now directly interact with patients, doctors and the whole medical team to provide services, so our job description has changed considerably. In this context, I think it is necessary to implement the CPS in all hospitals.” (P13)*

### Barriers Toward CPS

#### Insufficient Competency of Pharmacy Staff

Participants were asked about some obstacles encountered in the process of implementing the CPS. Some interviewees indicated that insufficient training of pharmacists, their poor clinical competence, and understaffing are currently the major problems.

'*Our biggest problem in China is that Chinese medical institutions do not train pharmacists very much. Our directors of pharmacy have insufficient skills and lack some management skills' (P15)*

“*Also, our financial support is not enough. Our current pharmacist team is understaffed and the structure is unreasonable. This system, including our continuing medical education, is not perfect, and our ability to serve our pharmacy is lagging behind.” (P14)*

#### Unclear Authority

Some participants believed that the CPS documents do not clearly define the responsibilities between the chief pharmacist and the director of the pharmacy department.

“*First of all, our authority is not clear, because there is a chief pharmacist in our hospital, then there is a director of the entire pharmacy department, then we have a general pharmacist committee in the hospital, of which I am the chairman. There are eight individuals, and the hospital did not clarify the division of job responsibilities to each person. The division of duties between the chief pharmacist and the director of the pharmacy department is unclear.” (P11)*

“*The levels of administration within the pharmacy department of a hospital were not covered in the document, and there was no precedent in the past. Therefore, the chief pharmacist and the former pharmacy director were treated in the same way and the level of administration of the chief pharmacist was not fully implemented.” (P2)*

“*I want to say that mainly the positioning of pharmacists within a department must be clear, and then the implementation will be good. Currently, it is very confusing.” (P7)*

#### Insufficient Salary

Some interviewees believed that the salary of pharmacists was generally low, especially for clinical pharmacists, which had affected the provision of better clinical pharmacy services.

“*We especially need clinical pharmacists, but clinical pharmacists do not belong directly to the pharmacy department. We do not pay them the salary. The bonus is also not issued from here, so he is not bound by us.” (P15)*

'*Then there are rewards and incentives. We must now ensure that our clinical pharmacists receive the highest bonus, so that each pharmacist has a goal, a career plan, and everyone should not fall behind.”(P7)*

#### Medical Insurance

Some interviewees thought that the implementation of the CPS required the support of relevant medical insurance policies.

“*Some of the problems we encountered in our work, as well as other experts mentioned, we also feel that the medical insurance payment system has to play a role so that the hospital pharmacy staff will have a high enthusiasm.” (P16)*

“*The sixth aspect is that we need the support of the medical insurance policy. It is also imperative.” (P18)*

### Suggestions Toward Chief Pharmacist Policy

#### Qualities to Become a Chief Pharmacist

Some participants believed that the appointment of a chief pharmacist is essential. The chief pharmacist should have relevant expertise and other abilities.

“*The third aspect is to let the hospital leadership accept this system properly. For example, let them learn from other institutions that have already set up the CPS, which is convenient for solving many problems encountered in work. The fourth aspect is that the appointed chief pharmacist must have considerable courage and considerable policy awareness, team management skills, and a sense of collaboration.” (P10)*

'*I strongly recommend the appointment of a chief pharmacist in the health administration department. For example, first, select a chief pharmacist in the municipal health administrative department and then get a core pharmacist in the province.'(P9)*

#### Incentives and Rewards

Many interviewees felt that rewards should be given to pharmacists who perform well to motivate pharmacists to work better.

“*It is necessary to highlight the working of pharmacy departments with the higher administration to let them understand the true value of the pharmacists.” (P1)*

“*In (the aspect of) hospital pharmacy department's job performance appraisal, the hospital should award the prize to the pharmacy department…... For the night shift, special positions, such as outpatient departments and other labor-intensive staff, reward the staff with good performance.” (P2)*

“*Supervise, manage, evaluate, and reward the use of drugs in clinical departments, and conduct special punish activity on the irrational use of drugs through inspection, and evaluation.” (P5)*

#### Proper Training

During the interview, different participants made different suggestions on the implementation of the CPS, and some of them suggested that pharmacists should be trained appropriately (Table 2).

**Table 2:** Key barriers and suggestions for implementation of Chief Pharmacist System.

**Barriers**
Insufficient competency of pharmacy staff
Unclear authority
Insufficient salary
Medical insurance
**Suggestions**
Qualities to become a chief pharmacist
Incentives and rewards
Proper training

“*We also propose that we should train the department leader to become a management-oriented cadre. Based on certain trainings, it can allow the candidate to match (the needs of) the position of the chief pharmacist.” (P10)*

*The government should vigorously support the development of the pharmacy team, create a platform for the development of the pharmacy team, unite the strength of the pharmacy team, and recognize the system and policies of the pharmaceutical service. (P10)*

“*Another point is that at the grassroots pharmacist education and training, we are doing this every year with this platform of the CPS.” (P12)*

## Discussion

This study aims to analyze the views of Chinese hospital pharmacists and government representatives on the implementation of the CPS under the background that the hospital pharmacy department had not been considered crucial for the healthcare system in China, for a long time. Previous published studies focused on evaluating the status of hospital pharmaceutical care services and clinical pharmacy research in China (55, 56). However, there is not much literature available on the newly implemented reform of the CPS in Chinese hospitals, which aims at extending the hospital pharmacy services beyond the mere procurement and distribution of drugs. The CPS focuses on the provision of pharmaceutical care and drug utilization evaluation under newly appointed/designated chief pharmacists (38). To our knowledge, this is the first study to reflect the status and effect of CPS by interviewing pharmacists and government staff.

In this study, most participants thought the chief pharmacist played a vital role in overall hospital services and they believed chief pharmacists had started to play a considerable role in many aspects of hospital pharmaceutical management, under the CPS. The chief pharmacist was responsible for the pharmaceutical management of the entire hospital and was primarily responsible for the rational use of medicines in the hospital, the improvement of the quality of pharmaceutical care services, the training of pharmacists, and the budget for drug procurement. The study results showed that after the implementation of the CPS, the situation of pharmacy consultations had improved under the leadership of the chief pharmacist. The hospital pharmacy department attached more attention to the prescription review and medication monitoring, and the management of medication had been improved. This reflects that the CPS can promote the rational use of drugs and improve the quality of pharmaceutical care services. These findings are in agreement with a previous study (38). All interviewees had a positive attitude toward the CPS. They believed that the implementation of the CPS can promote the transformation of the hospital's pharmacy department and allow pharmacists to enhance their prestige under the leadership of the chief pharmacist. This has already been reported in previous studies that hospital pharmacists can earn their desired prestige by engaging themselves in provision of pharmaceutical care services to the patients (57, 58). A recent study conducted in Kuwait found that chief pharmacists were willing to take part in patient centered roles including in provision of evidence-based medicine (59).

As the CPS has just been piloted, there are inevitably many obstacles and deficiencies in its implementation process. Many interviewees indicated that the policy did not clearly distinguish the powers and duties between the newly designated chief pharmacist and the director of the pharmacy department. This has made it difficult for chief pharmacists to play their roles effectively. The recognition of the chief pharmacist in the hospital was not high and they encountered difficulties in carrying out their duties. The main reason for this problem is that many people appointed as chief pharmacists were directors of the hospital's pharmacy department, so their powers had not changed after being appointed as the chief pharmacists. The government needs to provide a clear job description of the chief pharmacist about their duties and responsibilities with adequate training to help cope with this situation.

Problems such as lack of pharmacist ability and low enthusiasm due to low salary of the pharmacist were also encountered during the implementation of the CPS. This has been reported in previous studies that salary of pharmacists is one of the most integral motivator factors that could significantly impact the performance of pharmacists (60, 61). Therefore, it is recommended that salary of hospital pharmacists should be proportional to their amount of work and responsibility level (62).

Furthermore, the implementation of the relevant medical insurance policy could help hospitals better implement the chief pharmacist policy. For example, in 2019, China's National Healthcare Security Administration issued two key technical documents for a pilot project that introduces diagnostic related groups (DRG), which is a patient classification to standardize payment in national medical insurance schemes. Under the DRG model, patients will be classified into DRGs with similar clinical symptoms and resource costs on the basis of their age, gender, length of stay and clinical diagnosis. Thus, medical fees and insurance payments will be based on the DRG classification instead of specific patients, which can help prevent excessive treatment and overuse of medications and examinations (63, 64).

The interviewed experts suggested improving the abilities of the chief pharmacists with prior training. The selected chief pharmacist should not only have strong professional knowledge but also have excellent management ability, to better manage the hospital pharmacy team. There is also a need to motivate pharmacists who perform well, by providing financial and other incentives so that they can work better as highlighted in literature (65, 66). At the same time, it is necessary to strengthen pharmacist training, which will improve their ability to promote rational drug use within hospitals. Previous studies have shown that prior training of healthcare professionals could help them better understand the system which could offer positive outcomes (67, 68).

The CPS has many advantages and achieved some good results, but more efforts from the government, hospitals and pharmacists are needed to improve this system further, meet the real purpose of this system, and change the current situation of hospital pharmacy, provide better hospital pharmacy services to patients, and realize the actual value of pharmacists.

To the best of authors knowledge, this is an exploratory study which investigated the views of chief pharmacists, pharmacy heads, and government representatives regarding the CPS in various hospital settings of China. However, some limitations need to be noted. First, due to the inconsistent implementation time of CPS in different regions and the inconsistent documents formulated for CPS in the different areas, the interviewees' answers to the questions may vary in different regions and hospitals. To cover this problem, the interviewees who were included were all from pilot regions. Second, due to the small number of hospitals currently piloting this system in China, the number of chief pharmacists who could be interviewed was also small, and it may not be possible to systematically and comprehensively explain CPS. Third, this study only explored the perspectives of participants at the management level (head, chief, policymaker) on CPS and the views of other pharmacists are missing. Irrespective of all the above limitations, this study has provided useful insights about the different aspects of the CPS including its merits, barriers in its implementation, and suggestions from experts.

## Conclusion

In conclusion, the participants had a positive attitude toward CPS and CPS could help solve the current hospital pharmaceutical dilemma of being undervalued, improve the quality of hospital pharmaceutical care services, and promote rational drug use. Insufficient experience/training of pharmacy personnel, unclear authority, and inadequate salary are the major hurdles in the implementation of CPS in China. More efforts are needed to help implement CPS effectively in all hospitals across China.

## Data Availability Statement

The original contributions presented in the study are included in the article/supplementary material, further inquiries can be directed to the corresponding authors.

## Ethics Statement

The studies involving human participants were reviewed and approved by Ethics Committee of Xi'an Jiaotong University (number: 2019-1243). The patients/participants provided their written informed consent to participate in this study.

## Author Contributions

RY, QL, KH, and YF: conceptualization. CC and PL: data curation. RY, QL, and KH: formal analysis. YF: funding acquisition and supervision. WL and JW: investigation. QL, KH, WL, and JC: methodology. PZ: project administration. CC: software. QL and QD: writing—original draft. KH and YF: writing—review and editing. All authors contributed to the article and approved the submitted version.

## Funding

This work was funded by the Young Talent Support Plan, High Achiever Plan of Health Science Center, Xi'an Jiaotong University.

## Conflict of Interest

The authors declare that the research was conducted in the absence of any commercial or financial relationships that could be construed as a potential conflict of interest.

## Publisher's Note

All claims expressed in this article are solely those of the authors and do not necessarily represent those of their affiliated organizations, or those of the publisher, the editors and the reviewers. Any product that may be evaluated in this article, or claim that may be made by its manufacturer, is not guaranteed or endorsed by the publisher.
